# Novel Divisome-Associated Protein Spatially Coupling the Z-Ring with the Chromosomal Replication Terminus in Caulobacter crescentus

**DOI:** 10.1128/mBio.00487-20

**Published:** 2020-04-28

**Authors:** Shogo Ozaki, Urs Jenal, Tsutomu Katayama

**Affiliations:** a Department of Molecular Biology, Graduate School of Pharmaceutical Sciences, Kyushu University, Fukuoka, Japan; b Biozentrum, University of Basel, Basel, Switzerland; Max Planck Institute for Terrestrial Microbiology

**Keywords:** *Caulobacter crescentus*, cell division, chromosome organization, chromosome segregation, subcellular localization

## Abstract

Cell division requires proper spatial coordination with the chromosome, which undergoes dynamic changes during chromosome replication and segregation. FtsZ is a bacterial cytoskeletal protein that assembles into the Z-ring, providing a platform to build the cell division apparatus. In the model bacterium Caulobacter crescentus, the cellular localization of the Z-ring is controlled during the cell cycle in a chromosome replication-coupled manner. Although dynamic localization of the Z-ring at midcell is driven primarily by the replication origin-associated FtsZ inhibitor MipZ, the mechanism ensuring accurate positioning of the Z-ring remains unclear. In this study, we showed that the Z-ring colocalizes with the replication terminus region, located opposite the origin, throughout most of the C. crescentus cell cycle. Spatial organization of the two is mediated by ZapT, a previously uncharacterized protein that interacts with the terminus region and associates with ZapA and ZauP, both of which are part of the incipient division apparatus. While the Z-ring and the terminus region coincided with the presence of ZapT, colocalization of the two was perturbed in cells lacking *zapT*, which is accompanied by delayed midcellular positioning of the Z-ring. Moreover, cells overexpressing ZapT showed compromised positioning of the Z-ring and MipZ. These findings underscore the important role of ZapT in controlling cell division processes. We propose that ZapT acts as a molecular bridge that physically links the terminus region to the Z-ring, thereby ensuring accurate site selection for the Z-ring. Because ZapT is conserved in proteobacteria, these findings may define a general mechanism coordinating cell division with chromosome organization.

## Abstract (precis)

Growing bacteria require careful tuning of cell division processes with dynamic organization of replicating chromosomes. In enteric bacteria, ZapA associates with the cytoskeletal Z-ring and establishes a physical linkage to the chromosomal replication terminus through its interaction with ZapB-MatP-DNA complexes. However, because ZapB and MatP are found only in enteric bacteria, it remains unclear how the Z-ring and the terminus are coordinated in the vast majority of bacteria. Here, we provide evidence that a novel conserved protein, termed ZapT, mediates colocalization of the Z-ring with the terminus in Caulobacter crescentus, a model organism that is phylogenetically distant from enteric bacteria. Given that ZapT facilitates cell division processes in C. crescentus, this study highlights the universal importance of the physical linkage between the Z-ring and the terminus in maintaining cell integrity.

## INTRODUCTION

Coordinating cell division with chromosome replication and segregation is fundamental to the accurate transmission of genetic materials to offspring. In eukaryotes, cells sequentially undergo chromosome replication, segregation, and cell division at distinct cell cycle phases to ensure proper execution and completion of the events in time and space. In contrast, bacteria have the capacity to execute chromosome replication and segregation alongside cell division, thereby compressing cell cycle processes to achieve rapid growth (1). Therefore, to successfully transmit the chromosome to daughter cells over generations, bacteria require regulatory tuning of cell division processes with the chromosomal architecture, which undergoes dynamic changes during chromosome replication and segregation (2–5). However, chromosomal organization characteristics differ among various bacteria and it remains unclear how the system that coordinates cell division with dynamic chromosome architecture is conserved in the bacterial kingdom.

Bacterial cell division is initiated by the ubiquitous GTPase FtsZ (6–10), a tubulin homolog that polymerizes into bundled filaments termed Z-rings (11–13). The formation of the Z-ring at incipient division sites provides a scaffold for assembly of the divisome, a dynamic protein complex responsible for cell division (6, 14). Moreover, Z-rings can act as treadmilling platforms for the peptidoglycan synthesis machinery to stimulate septum formation (15, 16). To prevent the production of abnormal cells with no chromosomes or guillotined chromosomes, the position and timing of Z-ring formation must be tightly regulated in concert with the dynamic chromosomal architecture throughout the cell cycle. In the gammaproteobacterium Escherichia coli, at least two inhibitory systems operate to ensure Z-ring formation at the midcell region: Min and nucleoid occlusion. In the Min system, the FtsZ antagonist MinC shuttles dynamically between the two cell poles with the aid of MinD and MinE, thereby inhibiting Z-ring formation at the poles (17–21). In contrast, the nucleoid occlusion system relies on the nucleoid-associated FtsZ inhibitor SlmA to prevent Z-ring formation over the chromosomes (22–24). While neither *minCDE* nor *slmA* is essential for growth, inactivation of both leads to a synthetic lethal phenotype (25). Thus, the Min and nucleoid occlusion systems complementarily regulate Z-ring formation.

FtsZ-associated protein Z-ring-associated protein A (ZapA) is also implicated in spatial coordination of the Z-ring in E. coli (26). ZapA binds directly to FtsZ to stabilize the Z-ring at midcell (27). ZapA homologs are found in most bacterial species, suggesting that it plays an evolutionarily conserved role in Z-ring formation (14, 28, 29). In addition, ZapA forms a complex with the coiled-coil protein ZapB, which has a unique affinity for the nucleoid-associated MatP protein (27, 30, 31). MatP, a prerequisite for compaction of the replication terminus of the E. coli chromosome, binds to the 13-mer *matS* sites that are distributed within an 800-kb stretch around the replication terminus, a region opposite the origin of chromosome replication (32–34). Because the MatP-*matS* complexes can interact, bridging DNA between distal *matS* sites, MatP-mediated DNA clustering helps organize the replication terminus region into a compacted DNA called the Ter macrodomain (33, 35, 36). Thus, the interaction network comprising *matS*, MatP, ZapB, ZapA, and FtsZ, termed the Ter linkage, is proposed to connect the chromosome replication terminus with the Z-ring for regulatory tuning of Z-ring formation in time and space (9). Mutants lacking the *zapB* or *matP* gene are moderately compromised for chromosome segregation and cell division, highlighting the role of the Ter linkage in these cell cycle processes (30). Intriguingly, ZapB and MatP homologs are found only in enteric bacteria. Thus, the general relevance of the physical linkage between the chromosomal replication termini and the Z-ring outside the enteric bacterial species remains unknown.

**FIG 1:**
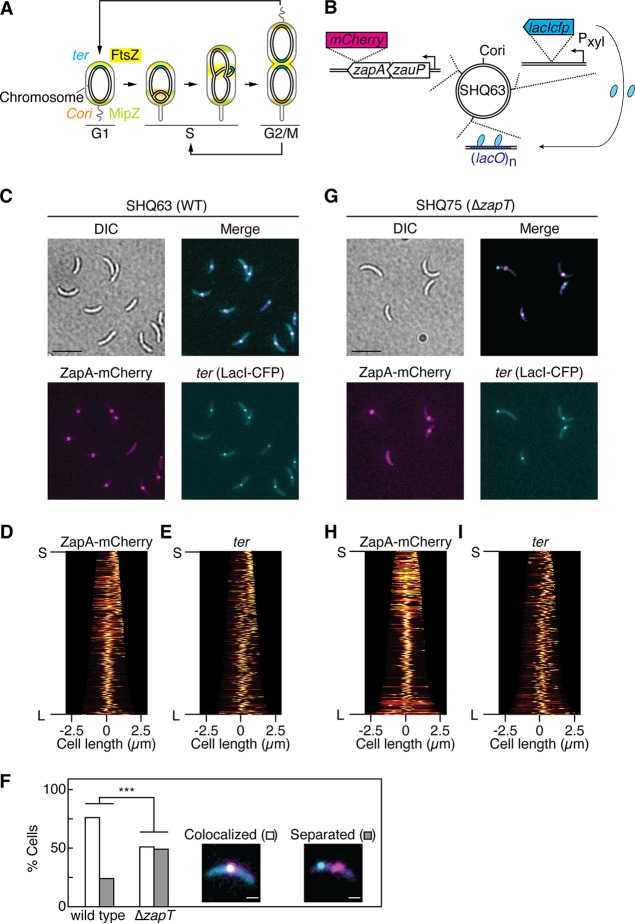
The replication terminus region of the C. crescentus chromosome colocalizes with the divisome in a ZapT-dependent manner. (A) The cell cycle of C. crescentus. Localizations of the origin of replication (Cori, orange), the terminus (*ter*, cyan), FtsZ (yellow), and MipZ (green) at distinct cell cycle stages (G_1_, S, G_2_/M) are shown schematically. (B) Schematic representation of FROS-ter, which consisted of three modules: LacI-CFP expressed from the xylose-dependent promoter (Pxyl), (*lacO*)n repeats integrated near the terminus, and a *zapA-mCherry* fusion replacing the native *zapA* gene. The relative positions of the individual modules are shown schematically on the circular C. crescentus genome. (C to I) Analyses of FROS-ter in wild-type and Δ*zapT* mutant strains. SHQ63 (wild-type [WT]) cells (C to E) and SHQ75 (Δ*zapT*) cells (G to I) grown in M2G medium were analyzed using fluorescence microscopy. Representative differential interference contrast (DIC) images and fluorescent images of ZapA-mCherry and LacI-CFP are shown. Scale bar (C and G), 5 μm. Demographs of ZapA-mCherry (D and H) and LacI-CFP (E and I) expression were generated using Oufti software. For cells with a unipolar ZapA-mCherry focus, the ZapA-marked cell pole was defined as a new pole. “S” and “L” indicate the shortest and longest cells, respectively. (F) The percentages of cells in which the distance between the LacI-CFP focus and ZapA-mCherry focus was ≤5 pixels were plotted as “colocalized” (*n* > 200). ***, confidence level of >99.9% by a z-test.

In this report, we provide evidence that the aquatic alphaproteobacterium Caulobacter crescentus, a model organism that is phylogenetically distant from enteric bacteria, physically links the Z-ring to the replication terminus region *in vivo*. This bacterium grows by asymmetric binary fission, producing two genetically identical but physiologically distinct progeny cells (37–41) (Fig. 1A). Whereas the stalked progeny is sessile and able to initiate chromosome replication and cell division (entry into the S phase), the motile swarmer progeny blocks replication initiation, thereby experiencing an extended nonproliferating period termed the G_1_ phase. Localization of C. crescentus FtsZ is subjected to cell cycle regulation primarily by the ParA-type ATPase MipZ, which directly binds FtsZ to destabilize its polymerization (42, 43). In the G_1_ phase, MipZ is sequestered to the old cell pole, located opposite the new cell pole that inherits FtsZ from the previous cell division. As the cell enters the S phase, MipZ adopts a bipolar localization, thereby forcing FtsZ to relocate to the midcell region, where the MipZ concentration is low. Here, we show that FtsZ colocalizes with the replication terminus region throughout most of the C. crescentus cell cycle. Moreover, we identified a Z-ring-associated protein that controls Z-ring colocalization with the terminus region (ZapT). Although ZapT was annotated as a putative transcriptional regulator belonging to the MerR protein family (44), potential ZapT binding sites are preferentially distributed within the open reading frames near the terminus-proximal regions. Moreover, we found that ZapT interacts with ZapA and ZauP, the counterpart of E. coli ZapA and the functional homolog of E. coli ZapB, respectively. Furthermore, we provide evidence that ZapT is an important component controlling cell division and chromosome segregation in C. crescentus. Because putative ZapT homologs are found in diverse proteobacterial species, the regulatory mechanism mediated by ZapT might be widely conserved.

## RESULTS

### The C. crescentus divisome colocalizes with the chromosome terminus.

To examine whether the terminus colocalizes with the divisome in C. crescentus, we used fluorescence-reporter-operator system-based terminus visualization (FROS-ter), in which a (*lacO*)n array integrated near the terminus (*ter*) region is visualized in living cells through its specific interaction with LacI-CFP (LacI-cyan fluorescent protein) (45). In addition, to monitor the position of the divisome simultaneously, a C-terminal mCherry fusion of ZapA (ZapA-mCherry), which colocalizes with the Z-ring throughout the cell cycle (14), was expressed from the native locus in the FROS-ter background (Fig. 1B).

In exponentially growing cell cultures, ZapA-mCherry and LacI-CFP appeared as a single focus in most cells (Fig. 1C). Demographic representations revealed that the ZapA-mCherry focus preferentially localizes at one cell polar region in shorter G_1_ cells and relocates to the midcell region as the cells grow (Fig. 1D). This is consistent with previous reports describing the subcellular dynamics of FtsZ and ZapA (14, 42). Moreover, we found that the demographic representation of ZapA-mCherry largely coincided with that of LacI-CFP (Fig. 1D and E), indicating that the *ter* region also exhibits a dynamic behavior akin to that of the divisome during the cell cycle. Further investigation of individual cells revealed that in a large proportion of cells (75%), the pixel distances between ZapA-mCherry and LacI-CFP foci were ≤340 nm (5 pixels) (Fig. 1D to F; see also Fig. S1 in the supplemental material). Together, these observations indicate that the divisome colocalizes with the *ter* region through most of the *Caulobacter* cell cycle. It should be noted that the ZapA-mCherry and LacI-CFP foci did not colocalize in a subpopulation (25%) of cells (Fig. 1F), implying that the distance between the divisome and the *ter* region can change dynamically within a certain time scale, possibly through association/dissociation cycles.

10.1128/mBio.00487-20.2 FIG S1 Localization of ZapA and *ter*. The pixel distance between the ZapA-mCherry centroid and the centroid of LacI-CFP that binds to the terminus region was measured using ImageJ, and the distribution is shown using a box plot. The *P* value was calculated using the Mann-Whitney-Wilcoxon test. Download FIG S1, PDF file, 0.8 MB. Copyright © 2020 Ozaki et al. 2020 Ozaki et al. This content is distributed under the terms of the Creative Commons Attribution 4.0 International license.

### Identification of ZapT.

To explore the molecular link between the divisome and the *ter* region, we set out to identify novel divisome components that interact directly with the *ter* region in C. crescentus. Guided by previous studies of the interaction between the divisome and the chromosome terminus in E. coli (9), we focused our attention on ZapA and its interaction partner ZauP (29). Their counterparts in E. coli, ZapA and ZapB, respectively, form a complex with MatP, a nucleoid-associated protein that binds to DNA loci near the chromosome terminus. Although MatP is conserved exclusively in *Enterobacteriaceae* and *Vibrionaceae* species (33), we reasoned that a functional homolog of MatP could operate in C. crescentus through a direct interaction with ZapA and/or ZauP.

**FIG 2:**
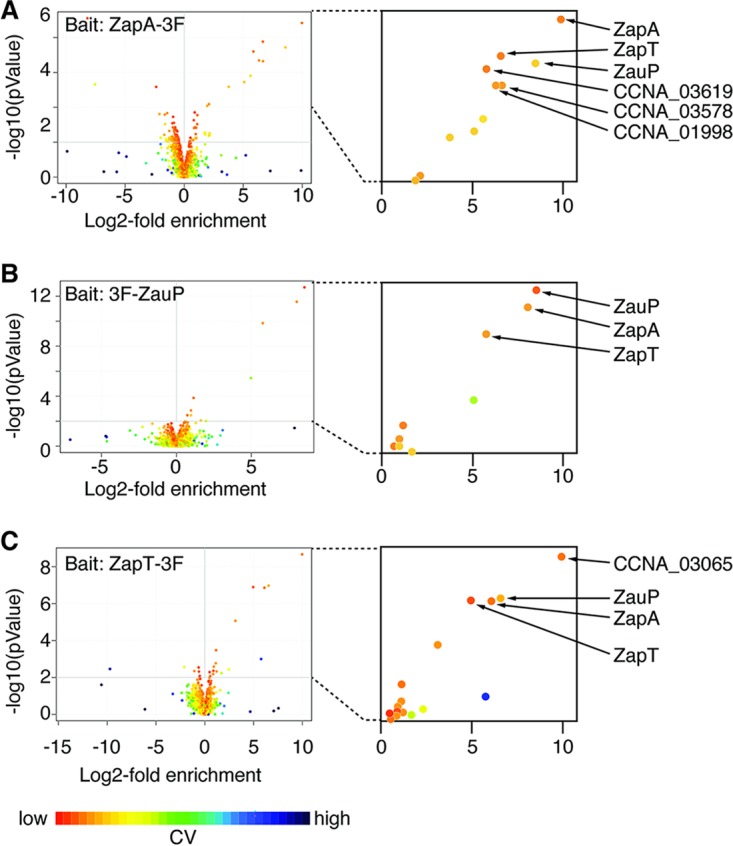
CoIP-MS analyses of ZapT, ZapA, and ZauP. Immunoprecipitation experiments were performed using the control NA1000 strain or strains expressing C-terminally 3xFLAG-tagged ZapA (ZapA-3F) (A), N-terminally 3xFLAG-tagged ZauP (3F-ZauP) (B), or C-terminally 3xFLAG-tagged ZapT (ZapT-3F) (C) and anti-M2 antibody-conjugated magnetic beads. Proteins retained on the beads were analyzed using mass spectrometry. Data obtained from three biological replicates are shown as volcano plots. CV, coefficient of variation.

To identify potential interacting partners of ZapA and ZauP, we used coimmunoprecipitation coupled to mass spectrometry (CoIP-MS) to examine a strain expressing either ZapA with a C-terminal 3xFLAG tag (ZapA-3F) or ZauP with an N-terminal 3xFLAG tag (3F-ZauP). The FLAG-tagged proteins in the cell lysate were recovered using anti-FLAG antibody-conjugated magnetic beads, and the materials retained on the beads were analyzed by mass spectrometry. To eliminate proteins that interacted nonspecifically with the anti-FLAG antibody or the beads, we performed similar experiments using the wild-type NA1000 strain as a negative control. Significant differences between the levels of protein enrichment in the FLAG-tagged samples and control samples were visualized using a volcano plot (Fig. 2A and B). The profiles revealed that ZauP, the putative DNA binding protein ZapT (CCNA_01434; annotated as a MerR-type transcriptional regulator) (Fig. S2A), a uridylate kinase (CCNA_01998), the RecQ DNA helicase (CCNA_03578), and a putative zinc metalloprotease (CCNA_03619) were highly enriched in the elution fraction of the strain expressing ZapA-3F (Fig. 2A). In addition, ZapA and ZapT were enriched in the elution fraction of the strain expressing 3F-ZauP (Fig. 2B), exposing ZapT as a likely candidate DNA binding protein associated with ZapA and ZauP.

10.1128/mBio.00487-20.3 FIG S2 Conservation of ZapT. (A) The secondary structure of ZapT was predicted using HHpred and the best-hit template MerR (PDB identifier [ID]: 5CRL). Identical residues are highlighted. (B) Alignment of putative ZapT orthologs. Proteins were aligned using the t-coffee algorithm (https://toolkit.tuebingen.mpg.de/tools/tcoffee). For conserved N- and C-terminal regions, the sequence logo is shown with the residue numbering of ZapT. Download FIG S2, PDF file, 1.5 MB. Copyright © 2020 Ozaki et al. 2020 Ozaki et al. This content is distributed under the terms of the Creative Commons Attribution 4.0 International license.

To corroborate this idea, we performed an additional CoIP-MS analysis using a strain expressing ZapT with a C-terminal 3xFLAG tag (ZapT-3F) from the native locus and found that both ZapA and ZauP were highly enriched in the elution fraction (Fig. 2C). These findings indicate that ZapT resides in close proximity to the Z-ring as a consequence of a direct or indirect interaction with ZapA and ZauP. It should be mentioned that our CoIP experiments failed to enrich FtsZ or other divisome components in the elution fraction, possibly because the protein interactions within the divisome are not very stable and thus were lost during the washing step in our assay. In contrast, ZapA, ZauP, and ZapT appeared to form a stable protein complex.

To investigate if ZapT is conserved in other bacteria, we performed the analysis of gene clusters in combination with gene cooccurrence. Because the C. crescentus genome encodes five MerR-family proteins (including ZapT), a general sequence-homology search failed to distinguish between ZapT homologs and homologs of other four MerR-family proteins. However, the *zapT* gene is arranged in an operon, with the upstream *ihfA* gene encoding integration host factor A (46). Thus, we reasoned that a MerR family protein that is encoded by a gene flanking the *ihfA* gene in a possible operon should be a putative ZapT homolog. Moreover, given that ZapT operates together with ZapA and ZauP, the putative *zapT* gene must coexist with *zapA* and *zauP* genes in the same genome. On the basis of these ideas, we surveyed the genomic sequences of the 22 organisms that were previously reported to possess putative *zapA* and *zauP* genes (29). Of these, four organisms do not carry an annotated *ihfA* gene. In contrast, all of the other organisms conserve the possible operon comprising the cognate *ihfA* gene and a putative *zapT* gene belonging to the MerR family (Fig. S2B). Those organisms include the alphaproteobacterium Brucella abortus, the betaproteobacterium Burkholderia rhizoxinica, and the gammaproteobacterium Pseudomonas aeruginosa. This observation is in line with the idea that ZapT homologs are widespread in diverse proteobacterial species.

### ZapT facilitates positioning of the Z-ring.

**FIG 3:**
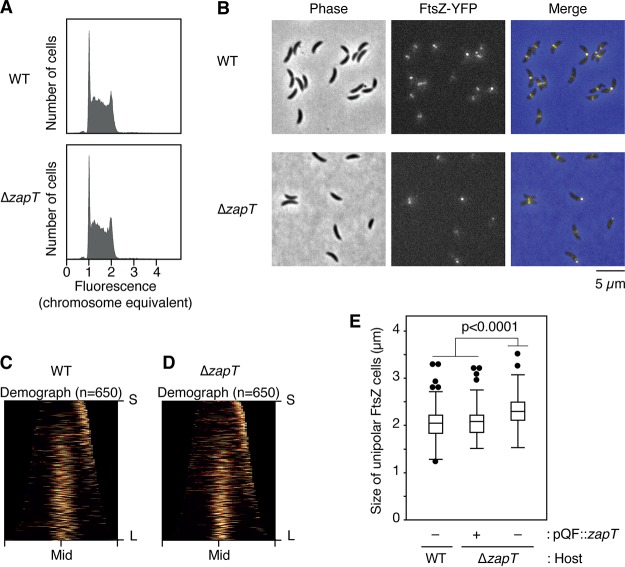
DNA content and FtsZ positioning in Δ*zapT* mutant cells. (A) The DNA contents of exponentially growing NA1000 (wild-type [WT]) and SHQ48 (Δ*zapT*) cells were analyzed using flow cytometry. For each strain, 50,000 cells were counted. (B to D) Localization of FtsZ-YFP in SHQ67 (wild-type [WT]) and SHQ136 (Δ*zapT*) cells grown exponentially in PYE medium. (B) After induction of FtsZ-YFP by treatment with 1 mM vanillate for 1 h, phase-contrast and fluorescent images were taken using fluorescence microscopy. (C and D) Demographs (C, WT; D, Δ*zapT*; *n* = 650 per strain) were generated as described in the legend for Fig. 1. For cells with a unipolar FtsZ-YFP focus, the FtsZ-marked cell pole was defined as a new pole. (E) Plasmid complementation test. Size distribution of cells with a unipolar FtsZ focus was analyzed for SHQ67 (WT) and SHQ136 (Δ*zapT*) strains harboring pQF::*zapT* or the empty vector pQF, and the results are shown as a box plot. The *P* value was calculated using the Mann-Whitney-Wilcoxon test.

To determine if ZapT contributes to Z-ring formation, we analyzed a C. crescentus mutant strain lacking ZapT. To minimize the risk of a polar effect upon gene disruption, an in-frame deletion (Δ*zapT*) was introduced into the *zapT* gene. As shown previously for Δ*zapA* and Δ*zauP* mutants (29), the resulting Δ*zapT* mutant was viable in peptone-yeast extract (PYE) medium and the synthetic M2G medium (minimal medium supplemented with 0.2% glucose), displaying a doubling time comparable with that of the wild-type NA1000 strain. Consistently, the size distribution of the Δ*zapT* mutant cells grown in PYE medium was comparable to that of wild-type cells (with mean lengths of 2.4 ± 0.67 μm for the wild type and 2.4 ± 0.77 μm for Δ*zapT* mutant). Moreover, a flow cytometry analysis showed that cellular DNA contents were indistinguishable between wild-type and Δ*zapT* mutant cells (Fig. 3A).

To visualize the Z-ring, an FtsZ-YFP (FtsZ-yellow fluorescent protein) fusion was ectopically expressed from the vanillate-dependent promoter in exponentially growing cells (42). As expected, a discrete YFP focus was formed in most wild-type cells (Fig. 3B). A demographic representation revealed that the cell cycle dynamics of FtsZ localization coincide well with that of ZapA-mCherry, namely, unipolar localization in shorter G_1_-phase cells and midcell localization in larger S-phase and G_2_/M-phase cells (Fig. 1; see also Fig. 3B and C). Similarly, the Δ*zapT* mutant strain produced shorter cells with unipolar Z-ring and longer cells with midcell Z-ring. However, analysis of the size distributions of those shorter cells with unipolar FtsZ revealed that the Δ*zapT* mutant cells were more elongated than the wild-type cells (Fig. 3E), indicating that the timing of FtsZ relocation from the cell pole to midcell was slightly delayed in the Δ*zapT* mutant strain. The observed phenotype of the Δ*zapT* mutant was suppressed by the plasmid carrying *zapT* (Fig. 3E). Taken together with a previous report of the finding that a mutant Δ*zapA* mutant or Δ*zauP* mutant displayed moderately impaired midcell localization of the Z-ring (29), our findings indicate that, like ZapA and ZauP, ZapT is important for the timely formation of the Z-ring at midcell *in vivo*.

### ZapT ensures colocalization of the divisome with the *ter* region.

To further investigate whether ZapT facilitates colocalization of the divisome with the *ter* region, the *zapA-mCherry* allele and FROS-ter were introduced into the *ΔzapT* mutant strain. As seen in the wild-type *zapT* strain, ZapA-mCherry and LacI-CFP formed single foci in most cells (Fig. 1C and G). However, a demographic analysis revealed that the ZapA-mCherry signals were less concentrated at the cell poles in the shorter Δ*zapT* mutant cells than in the wild-type cells (Fig. 1D and H). A more erratic polar localization of ZapA-mCherry in the absence of ZapT is consistent with the observation that Z-ring formation at midcell was delayed in the Δ*zapT* mutant strain. Furthermore, colocalization of ZapA-mCherry and LacI-CFP was significantly reduced in Δ*zapT* cells (Fig. 1F; see also Fig. S1), suggesting that ZapT is required for robust colocalization of the *ter* region with the divisome. In a subpopulation of Δ*zapT* mutant cells, the position of ZapA-mCherry coincided with that of LacI-CFP, which might have been due to an accidental overlap of the two foci during random motion. Alternatively, an additional, as-yet-unidentified complementary pathway(s) might exist to ensure colocalization of the *ter* region with the divisome.

### Subcellular dynamics of ZapT.

**FIG 4:**
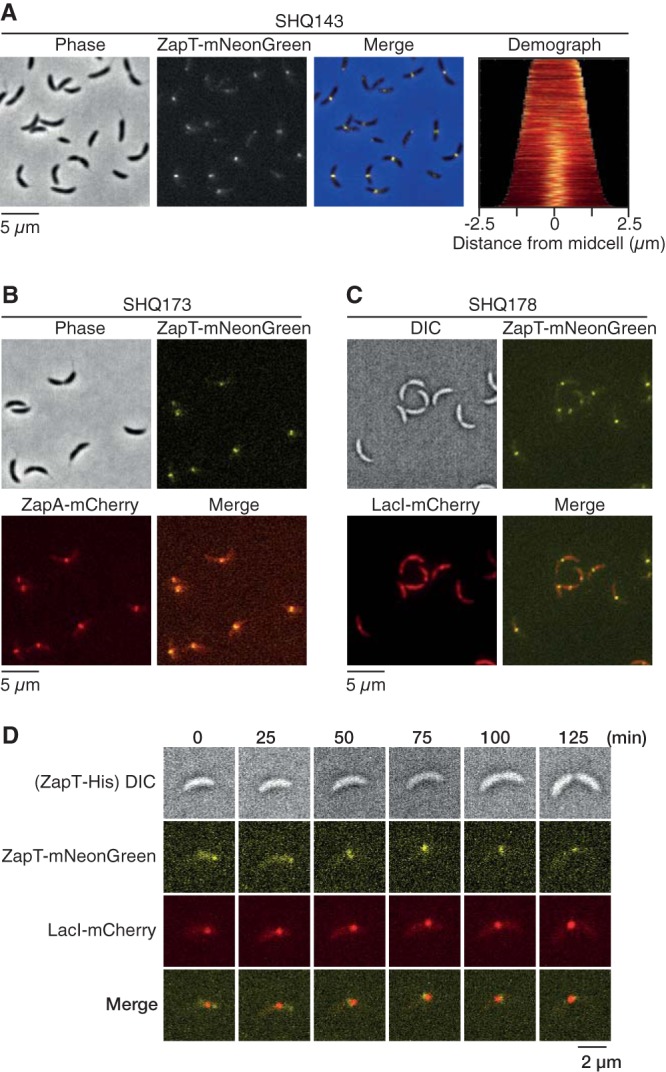
Subcellular positioning of ZapT. (A) Localization of ZapT-mNeonGreen in SHQ143 cells grown exponentially in PYE medium. Demographs were generated as described in the legend for Fig. 1. (B) Localization of ZapT-mNeonGreen and ZapA-mCherry in SHQ173 cells grown exponentially in PYE medium. Representative images were shown. (C and D) Localization of ZapT (mNeonGreen) and the terminus region (LacI-mCherry) in SHQ178 cells grown exponentially in PYE medium. Snapshot images (C) and time-lapse images (D) were shown.

To examine subcellular localization of ZapT, we generated a strain expressing a C-terminal mNeonGreen fusion of ZapT (ZapT-mNeonGreen) from the native *zapT* locus. Fluorescence microscopy of the resulting strain revealed that the localization pattern of ZapT-mNeonGreen parallels that of ZapA-mCherry: i.e., the cells formed a single discrete focus of ZapT-mNeonGreen at one cell polar region in shorter cells and at the midcell region in larger cells (Fig. 4A). Indeed, when ZapT-mNeonGreen and ZapA-mCherry were simultaneously expressed, the two positions coincided in most cells (Fig. 4B), supporting the idea that ZapT forms a complex with ZapA *in vivo*.

Next, we analyzed a possible interaction between ZapT and the *ter* region using a strain with the *zapT*-*mNeonGreen* allele in the FROS-ter background. As observed for ZapA (Fig. 1), ZapT-mNeonGreen formed a discrete focus at a position close to the *ter* region marked by the LacI-mCherry (Fig. 4C). Moreover, the time-lapse analysis demonstrated that ZapT-mNeonGreen undergoes dynamic changes in its location during the cell cycle, following the position of the *ter* region (Fig. 4D). These observations are consistent with the idea that the colocalization between divisome and the ter region is mediated by their interaction with ZapT.

### ZapT preferentially associates with the *ter* region.

**FIG 5:**
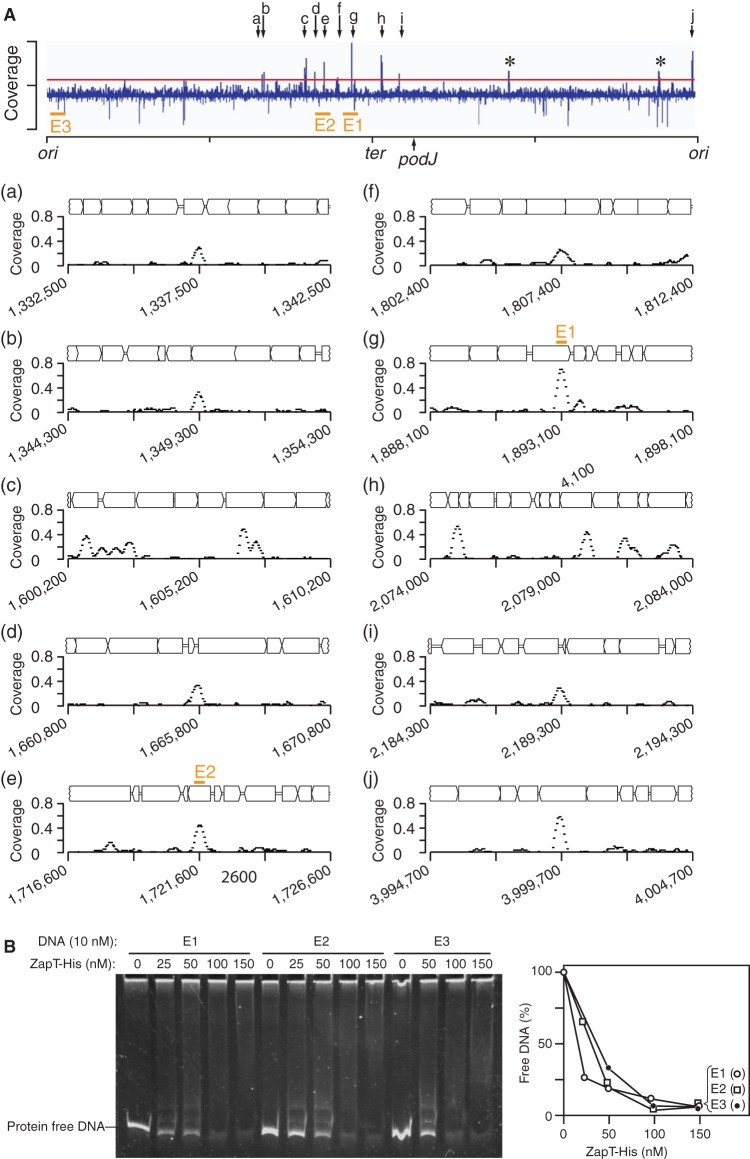
Genome-wide identification of ZapT binding sites. (A) ChIP-seq was performed using the SHQ10 (ZapT-3F) strain. The coverage of every 50-bp window was normalized to the total number of reads and plotted against the NA1000 genomic position. Asterisks indicate false-positive peaks that appeared in both the SHQ10 and control NA1000 samples. The horizontal red line indicates the threshold. The genomic positions that displayed distinct ChIP peaks in SHQ10 cells but not NA1000 cells are indicated by arrows a to j. Close-up representations of the individual ChIP peaks are also shown with gene arrangements. For simplicity, the names of the genes have been omitted. The *x* axis indicates the genomic position of the reference NA1000 genome. The origin (*ori*), terminus (*ter*), and the *podJ* locus used for the FROSter assay are indicated. (B) EMSA. The ligand DNA (E1, E2, or E3) (10 nM) was incubated with the indicated amounts of ZapT-His, followed by polyacrylamide gel electrophoresis. DNA was visualized by GelStar staining. The band signals of the protein-free DNA were analyzed using Image J, and the results are plotted as percentages.

Next, we analyzed the distribution of ZapT binding sites throughout the C. crescentus chromosome. We reasoned that ZapT binding sites are enriched within the *ter*-proximal region if ZapT plays a direct role in spatial coordination of the divisome and the chromosome terminus. To test this idea, we performed chromatin immunoprecipitation coupled to deep sequencing (ChIP-seq) using the ZapT-3F-expressing strain used for CoIP-MS. To eliminate false-positive signals, similar experiments were performed using the wild-type NA1000 strain as a negative control. On the basis of the ChIP-seq experiments, we identified 10 genomic positions that displayed discrete peaks of sequencing coverage in a ZapT-3F-dependent manner, a hallmark of ZapT binding sites (Fig. 5A). Although we did not find a consensus DNA sequence motif, these potential binding sites were mostly found within open reading frame regions (Fig. 5A). Moreover, the distribution of the ZapT binding sites was strongly biased toward the *ter*-proximal region. With one exception, the other binding sites were located at genome positions ranging from 1.3 to 2.2 MB, which corresponds to the chromosome terminus and its flanking regions. This finding suggests that ZapT-DNA complexes are preferentially generated at the *ter* region.

To probe for ZapT-DNA interactions *in vitro*, we performed an electrophoretic mobility shift assay using a C-terminally hexahistidine-tagged ZapT-3F protein (ZapT-His) and a 0.3-kb DNA fragment containing the highest ChIP peak, “g” (ligand E1); the intermediate peak “e” (ligand E2); or no distinct peak (ligand E3) (Fig. 5A; see also Fig. S3). In our experimental setup, ZapT-His retains the avidity to bind DNA, as the levels of the signals of the protein-free DNA decreased in a ZapT-His-dependent manner (Fig. 5B). The resulting DNA-ZapT-His complexes were not stable enough to form a discrete band. Also, the concentrations of ZapT-His required for a 50% decrease of the protein-free DNA were largely comparable for different DNA molecules tested, arguing for low sequence specificity of ZapT-His in DNA binding under *in vitro* conditions. Given that DNA binding proteins of the MerR family often require coeffectors to coordinate their DNA binding activities (44), it is conceivable that ZapT also relies on an unidentified factors mediating its interaction with the terminus region. Moreover, DNA topology such as DNA bending, looping, or superhelicity might be required for the specific interaction of ZapT with the terminus region.

10.1128/mBio.00487-20.4 FIG S3 Purification of ZapT-His. ZapT-His expressed in Rosetta 2(DE3) cells was purified through nickel-Sepharose and HiTrap heparin chromatography. The purity of ZapT-His in the final fraction was over 90%, as judged by the densitometric scanning. IPTG, isopropyl β-d-1-thiogalactopyranoside. –, whole cells before IPTG induction. +, whole cells after IPTG induction. Download FIG S3, PDF file, 0.3 MB. Copyright © 2020 Ozaki et al. 2020 Ozaki et al. This content is distributed under the terms of the Creative Commons Attribution 4.0 International license.

### Overproduction of ZapT blocks cell division.

**FIG 6:**
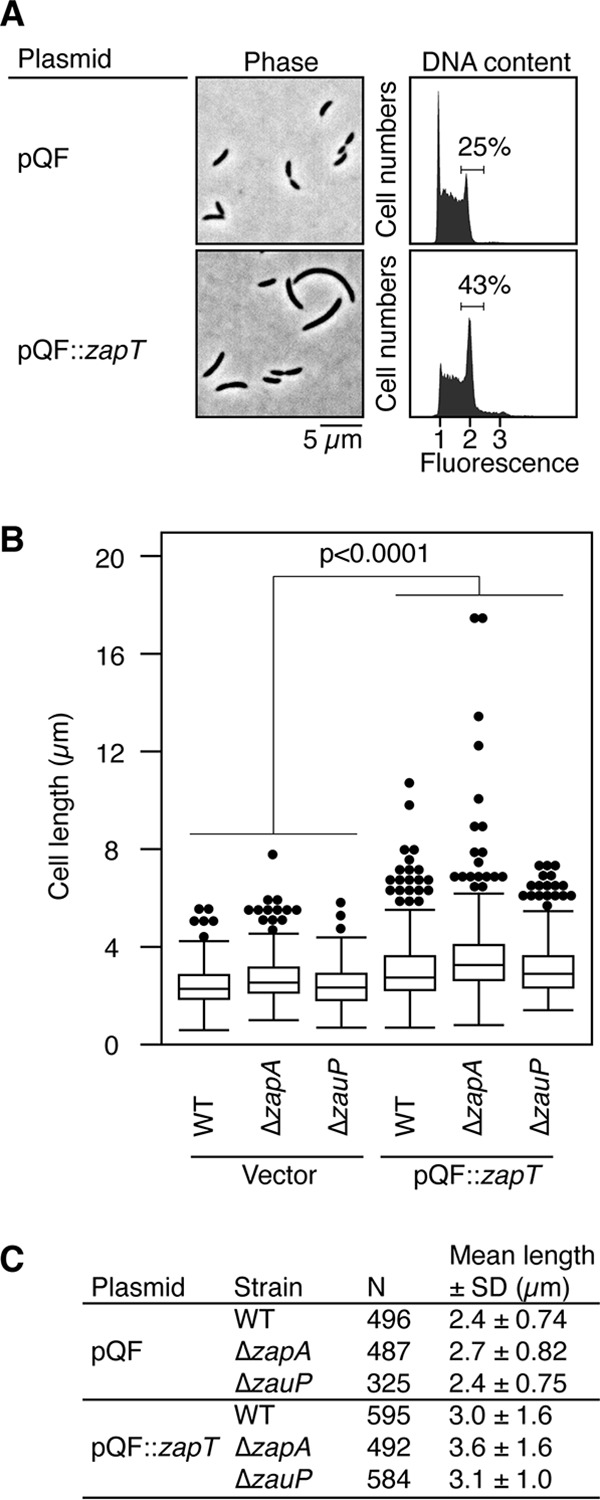
Overproduction of ZapT blocks cell division. The effect of overexpression of ZapT on the cell morphology and DNA content of the wild-type C. crescentus strain was assayed. The NA1000 strain carrying an empty vector (pQF) or its derivative harboring *zapT-3F* (pQF::*zapT*) was grown in PYE medium supplemented with 1 μM cumate for 6 h. Cell morphology and DNA content were analyzed using phase-contrast microscopy and flow cytometry, respectively. The number of cells containing two chromosome equivalents is shown as a percentage of the total number of cells. (B and C) The pQF plasmid or the pQF::*zapT* plasmid was introduced into the wild-type, Δ*zapA*, or Δ*zauP* strain. After a 6-h induction performed with 1 μM cumate, cells were analyzed using phase-contrast microscopy. (B) The distributions of cell lengths are shown using a box plot. The *P* value was calculated using the Mann-Whitney-Wilcoxon test. (C) The number of cells analyzed (N) and mean cell length with standard deviation (SD) are indicated.

To gain insight into the function of ZapT, we analyzed cell morphology and DNA content following overexpression of ZapT. To this end, we transformed the *Caulobacter* wild type with a low-copy-number plasmid expressing ZapT-3F from a cumate-dependent promoter or its vector control. After a 6-h induction with cumate, approximately 10-fold-excess amounts of ZapT-3F were accumulated (Fig. S4). Under these conditions, the wild-type *Caulobacter* cells carrying the ZapT-3F plasmid were more elongated than those carrying the empty vector (Fig. 6A). Indeed, the size distribution of ZapT-expressing cells was significantly differentiated from that of control (Fig. 6B and C). A flow cytometry analysis revealed that the fractions of cells harboring two chromosomes were markedly increased upon ZapT-3F induction (25% for vector control and 43% for the ZapT-3F plasmid) (Fig. 6A), arguing for a cell cycle delay in the G_2_/M phase. As a control, we performed similar experiments using overexpression of green fluorescent protein (GFP), which resulted in no obvious cell division delay (data not shown). These findings suggest that overexpression of ZapT interferes with the cell division process.

10.1128/mBio.00487-20.5 FIG S4 Overexpression of ZapT from a cumate-dependent promoter. NA1000 and SHQ10 cells harboring pQF or pQF::*zapT* or neither were grown in PYE medium supplemented with tetracycline and various concentrations of cumate (0, 0.0013, 0.0064, 0.032, 0.16, 0.8, 4, 20, or 100 μM [A] or 0, 0.1, 0.2, 0.4, 0.6, 0.8, 1, or 100 μM [B]) for 6 h, followed by Western blotting performed using an anti-Flag antibody (Wako). To deduce relative ZapT levels, the band intensities were analyzed using Image J software and normalized to those determined for ZapT-3F expressed from the chromosome copy. (C) NA1000 cells harboring the pQFzapTmNG plasmid were grown at 30°C for 6 h in PYE medium supplemented with tetracycline and 1 μM cumate, followed by fluorescence microscopy. Representative snapshot images are shown. Download FIG S4, TIF file, 1.9 MB. Copyright © 2020 Ozaki et al. 2020 Ozaki et al. This content is distributed under the terms of the Creative Commons Attribution 4.0 International license.

To investigate whether the observed cell division block was dependent on *zapA* or *zauP*, we performed a similar cell morphology analysis using a mutant strain harboring the Δ*zapA* or Δ*zauP* allele. The size distributions of cells expressing the empty vector were comparable for the wild-type, Δ*zapA*, and Δ*zauP* strains (Fig. 6B and C). In contrast, when ZapT-3F was overexpressed, the Δ*zapA* and Δ*zauP* strains produced elongated cells such as were observed in the wild-type strain background (Fig. 6B and C). Thus, the interference of ZapT overexpression with the cell division process occurs independently of ZapA or ZauP. It should be mentioned that a *zapA* mutant strain with an in-frame deletion of the residues between positions 13 and 97 was reported to produce mildly elongated cells (the mean length of 3.10 ± 1.90 μm), indicative of a cell division defect (29). Because the Δ*zapA* allele used in this study lacks the residues between positions 4 and 105, different 3′ ends of the *zauP* mRNA may explain differences in the cell division phenotype of these *zapA* mutants.

### Excess ZapT impairs midcell repositioning of the Z-ring.

**FIG 7:**
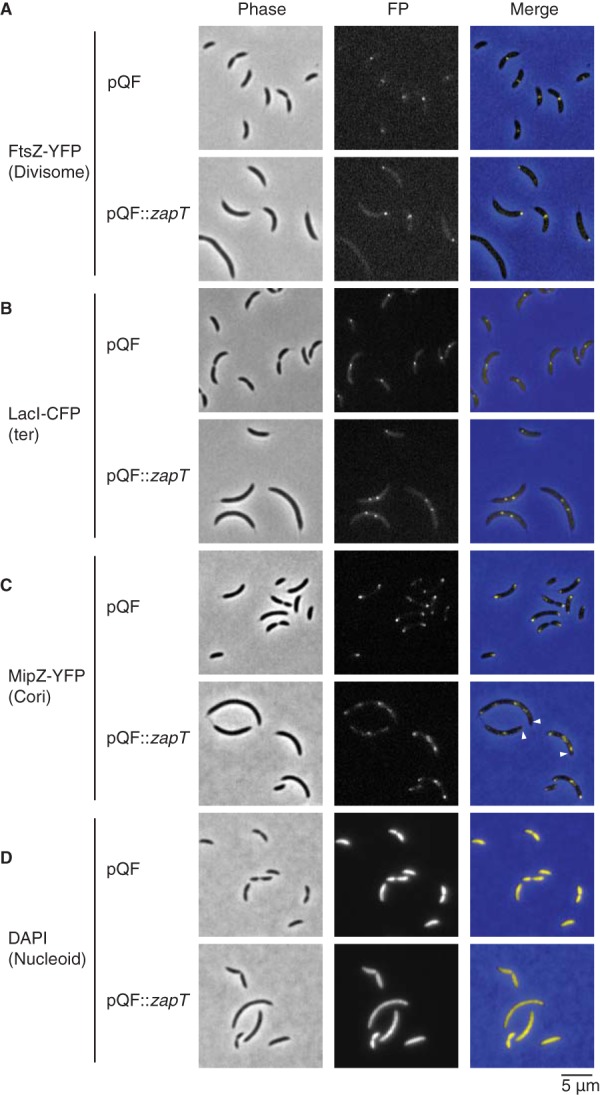
Overproduction of ZapT causes aberrant localization of FtsZ and MipZ. The images show localization of FtsZ, the terminus region, MipZ, and the nucleoid in the presence of excess ZapT. The pQF or pQF::*zapT* vector was introduced into a strain expressing FtsZ-YFP (SHQ67) (A), FROS-ter (SHQ54) (B), MipZ-YFP (SHQ66) (C), or none of those products (NA1000) (D). Log-phase cells that were incubated for 6 h in PYE medium supplemented with 1 μM cumate were analyzed by fluorescence microscopy. (D) For visualization of nucleoid, cells fixed in 70% ethanol were incubated for >10 min in 2 μg/ml DAPI solution before imaging. Arrowheads indicate polar MipZ-free zones.

To examine if the compromised cell division caused by excess ZapT-3F resulted from impaired positioning or assembly of the Z-ring, we analyzed the localization of an FtsZ-YFP fusion in cells overexpressing ZapT-3F (Fig. 7; see also Fig. S5). When ZapT-3F was overexpressed from a low-copy-number plasmid, the cells were elongated but retained the ability to form discrete YFP foci (Fig. 7A, lower panel), indicating that the presence of excess ZapT does not lead to general destabilization of FtsZ polymers. However, a large (50%) fraction of cells overproducing ZapT-3F displayed a unipolar FtsZ-YFP focus (Fig. 7A, lower panel). In contrast, in cells carrying the control vector, FtsZ-YFP localized mainly to midcell and only a small (10%) fraction of cells showed unipolar localization (Fig. 7A, upper panel). FtsZ localizes at midcell during most of the cell cycle to initiate cell division and transiently remains at the new cell pole after completion of division until relocating to midcell for the next round of cell division. Thus, the observed increase in unipolar localization of FtsZ-YFP in cells overexpressing ZapT-3F could be explained by debilitated or delayed relocation of FtsZ from the pole to midcell.

10.1128/mBio.00487-20.6 FIG S5 Demographic analysis for FtsZ-YFP, LacI-CFP, and MipZ-YFP. Related to Fig. 7, localizations of FtsZ, the terminus region, and MipZ in the presence or absence of excess ZapT were analyzed demographically. Demographic representations were generated as described in the legend for Fig. 1. Download FIG S5, PDF file, 2.8 MB. Copyright © 2020 Ozaki et al. 2020 Ozaki et al. This content is distributed under the terms of the Creative Commons Attribution 4.0 International license.

To examine if excess ZapT-3F also perturbs midcell repositioning of the *ter* region, FROS-ter experiments were performed in exponentially growing cells overexpressing ZapT-3F. Both the control strain carrying the empty vector and the strain overexpressing ZapT-3F produced cells with either one or two LacI-CFP foci (Fig. 7B). For the control vector strain, the localization profile of LacI-CFP was essentially the same as that observed earlier using wild-type cells (Fig. 7B, upper panel; see also Fig. 1): cells with a single focus near midcell (82%) or the cell pole (10%) and cells with two foci (8%) at midcell. In contrast, in a strain overexpressing ZapT, only 22% of the cells had a single CFP focus at midcell, while an increased number of cells showed two or three LacI-CFP foci (56% and 12%, respectively) (Fig. 7B, lower panels). Cells with two LacI-CFP foci were substantially elongated, and the foci were generally positioned slightly off midcell, where the *ter* region is sequestered after constriction in unperturbed cells. Moreover, we observed a small (11%) proportion of cells displaying unipolar localization of LacI-CFP in the ZapT-overexpressing strain, which was comparable to that observed for the control strain (8%). These observations are consistent with the idea that relocation of the terminus from the cell pole is largely maintained under conditions in which ZapT-3F is overexpressed although the positioning of the terminus at midcell is disturbed. Therefore, we suggest that excess ZapT specifically blocks midcell localization of the divisome without affecting the dynamics of the terminus.

To further interrogate the cause of the impaired positioning of FtsZ under conditions of excess ZapT, we turned our attention to MipZ, a ParA-type ATPase that inhibits FtsZ polymerization. Newborn *Caulobacter* cells sequester MipZ to the old cell pole, opposite the FtsZ-occupied new pole. Conversely, cells that are replicating their chromosomes impose bipolar localization on MipZ, which drives FtsZ relocation to midcell, where MipZ concentrations are low (42, 43). Because relocation of FtsZ from the cell pole to midcell is compromised in cells overexpressing ZapT-3F, we reasoned that excess ZapT might interfere with the bipolar localization of MipZ. To test this hypothesis, we analyzed localization of a C-terminal YFP fusion of MipZ (42). As expected, MipZ-YFP was localized at either one or both cell poles in the vector control strain (Fig. 7C). The fraction of cells with bipolar localization of MipZ-YFP (86%) was comparable with the fraction with midcell localization of FtsZ-YFP (90%) (Fig. 7A and C), which is consistent with the idea that the bipolar MipZ gradient restricts Z-ring formation near the midcell region. Next, we performed a similar experiment using cells overexpressing ZapT-3F. Overall, 96% of the cells overexpressing ZapT-3F formed two or more discrete MipZ foci and 4% formed a single focus, indicating that MipZ assembly was not affected by excess ZapT. Notably, in 50% of the cells harboring multiple MipZ-YFP foci, at least one of the foci was positioned distal to the cell pole, leaving polar zones devoid of MipZ. This finding is consistent with the observation that relocation of FtsZ is perturbed under conditions of excess ZapT. Because the entire cytosolic space was occupied by the nucleoid in a manner irrespective of ZapT overexpression (Fig. 7D) and overexpression of ZapT-mNeonGreen yielded cells with a single discrete fluorescent focus (Fig. S4C), mislocalization of MipZ is unlikely to result from the presence of a DNA-free zone at the cell pole or from the irregular condensation of the nucleoids. Also, a Δ*zapT* mutant grown in PYE complex medium showed normal MipZ localization (Fig. S6). Thus, an excess of ZapT changes the spatial arrangement of MipZ, either directly or indirectly.

10.1128/mBio.00487-20.7 FIG S6 Localization of MipZ. SHQ66 (A) and SHQ72 (B) cells grown exponentially in PYE medium at 30°C were analyzed using fluorescence microscopy. Representative images are shown. Demographs were generated as described in the legend for Fig. 1. For cells with a unipolar MipZ-YFP focus, the MipZ-marked cell pole was defined as the old pole. Download FIG S6, PDF file, 2.9 MB. Copyright © 2020 Ozaki et al. 2020 Ozaki et al. This content is distributed under the terms of the Creative Commons Attribution 4.0 International license.

## DISCUSSION

In this study, we found that the replication terminus of the *C. crescenuts* chromosome is physically linked to the divisome during most of the cell cycle. We propose that ZapT, a previously uncharacterized MerR family protein, contributes to spatial coordination of the terminus and divisome via its interaction with the Z-ring-associated proteins ZapA and ZauP. ZapT coincides with both ZapA and the terminus DNA in living cells. Notably, mutants lacking the *zapT* gene displayed misplacement of the divisome and the terminus, arguing that ZapT plays a prominent role in the physical link between these structures. The divisome and terminus are centrally colocalized in stalked and predivisional cells and, after division, remain localized at the new cell poles for a defined period (47–49). Thus, the physical linkage of the divisome and terminus appears spatially coordinated while cells progress through the cell cycle.

The observation that Z-ring formation at midcell is delayed in Δ*zapT* cells strongly argues for functional importance of ZapT in the process of cell division. In the absence of ZapT, G_1_ cells often failed to properly localize ZapA to the cell pole. Because ZapA was previously shown to facilitate Z-ring formation *in vivo* (29) and to interact with ZapT, the observed delay of formation of the Z-ring in the Δ*zapT* mutant cells likely resulted from irregular positioning of ZapA. The idea of a physiological role of ZapT in the process of cell division is further supported by the observation that excess ZapT levels caused a division arrest. Although the division-arrested cells retained the ability to recruit the chromosome terminus near midcell, the terminus had lost the physical connection to the divisome, leaving inert FtsZ polymers at the cell pole. In agreement with this, the FtsZ inhibitor MipZ was often mislocalized under these conditions, creating MipZ-free cell poles where FtsZ is able to polymerize. Therefore, it is possible that excess ZapT molecules might directly or indirectly interfere with MipZ to reduce its accessibility to the cell pole, thereby stabilizing polar FtsZ polymers. The polar localization of MipZ is driven by the essential DNA partitioning ATPase ParA that localizes to the FtsZ-marked new cell pole (50, 51). Because ZapT and ParA are sequestered to the same cell pole in the G_1_ phase, it is possible that the mislocalization of MipZ under ZapT overexpression results from perturbed functions of ParA. Coincidently, similar mislocalization of MipZ was observed in *Caulobacter* cells expressing the dominant-negative ParA K20R variant (52). The ParA Lys20 residue comprises the Walker A-type ATP binding motif. Thus, it is conceivable that overexpressed ZapT might directly or indirectly perturb the ATPase cycle of ParA, thereby inhibiting the process of MipZ localization. Under laboratory conditions, the mRNA levels of the *Caulobacter ihfA*-*zapT* operon peak in G_1_ phase and remain relatively low during the S phase where MipZ dynamically relocates to both poles (53, 54). Therefore, it is also possible that the observed impact of excess ZapT on MipZ localization might have been due to the overruling of physiological fluctuations of ZapT during the cell cycle, where differences in ZapT concentrations may facilitate spatial distribution of factions involved in cell cycle progression and cell division.

We propose that DNA-ZapT complexes play a direct role in the spatial coordination of the terminus and divisome. In E. coli, the nucleoid-associated MatP protein and the divisome component ZapB have been proposed to play a central role in maintaining a physical linkage between the terminus and divisome (33). Several lines of evidence indicate that MatP oligomers facilitate condensation of the replication terminus region of the E. coli chromosome through binding to multiple recognition sites distributed within an 800-kb stretch around the terminus (32–34). On the other hand, ZapB associates with FtsZ via ZapA (29). Thus, a physical connection of the terminus region to the Z-ring can be mediated by the physical interaction between MatP and ZapB. By analogy, it is possible that ZapT can form a nucleoprotein complex consisting of the terminus region of the C. crescentus chromosome, ZauP, and ZapA to recruit the Z-ring. This idea is fully consistent with our genome-wide ChIP-seq results indicating *ter-*proximal region preferential DNA binding of ZapT, although E. coli MatP shares little or no sequence similarity with C. crescentus ZapT.

In the colocalization analysis performed for ZapT and the terminus DNA, some cells form a ZapT-mNeonGreen focus at a position slightly distant from the *ter*-proximal *podJ* locus marked by LacI-mCherry (Fig. 4C and D). Because the ZapT binding sites determined by ChIP-seq are >90 kb away from the *podJ* locus (Fig. 5A), a spatial lag between ZapT-mNeonGreen and LacI-mCherry foci could be explained by the spacing between their binding sites. Also, it is possible that ZapT might primarily bind to the Z-ring rather than DNA. We infer that Z-ring-associated ZapT dynamically follows the movement of the terminus DNA with a certain lag time, which could reflect the distance between ZapT-mNeonGreen and LacI-mCherry foci.

Coordinating cell cycle progression with dynamic chromosome architecture is a sophisticated strategy to ensure the faithful inheritance of genetic materials. We suggest that the ZapT-ZapA-ZauP complex forms a physical linkage between the Z-ring and the chromosomal replication terminus. As ZapT, ZapA, and ZauP are conserved in various organisms, including members of the alpha-, beta-, and gammaproteobacterial groups, our findings expand our understanding of how cell division is coupled to chromosome replication and segregation in the bacterial kingdom. FtsZ and ZapA homologs are found even in the eukaryotic red alga Cyanidioschyzon merolae (FtsZ1 and ZED, respectively) and play an essential role in the cognate mitochondrial division (55–57). It is possible that a ZapT-like DNA binding protein could exist in this case, coordinating mitochondrial DNA with division.

## MATERIALS AND METHODS

### Bacterial strains and DNAs.

The strains, plasmids, and oligonucleotides used in this study are listed in Tables 1, 2, and 3, respectively. *Caulobacter* strains were grown at 30°C in PYE or minimal medium supplemented with 0.2% glucose (M2G). When necessary, the culture medium was supplemented with xylose (0.03%), cumate (1 μM), or vanillate (0.5 mM) as indicated. Detailed procedures for construction of the strains and plasmids are described in Text S1 in the supplemental material.

**TABLE 1:** Bacterial strains used in this study

Species and strain	Genotype	Reference or source
Caulobacter crescentus		
NA1000	A wild-type Caulobacter crescentus strain	61
PV2865	CB15N *cc0006*::(*tetO*)*n xylX*::*pHPV472 podJ*::*pHPV489*	45
SHQ10	NA1000 *CCNA_01434-3xFLAG*	This study
SHQ48	NA1000 Δ*zapT*	This study
SHQ56	NA1000 *zapA*::*mCherry*	This study
SHQ63	SHQ56 *xylX*::pHPV472 *podJ*::*pHPV489*	This study
SHQ66	NA1000 *mipZ*::pMT151(*PmipZ*::*mipZyfp Pxyl*::*mipZ*)	This study
SHQ67	NA1000 *vanA*::pMT383(*ftsZyfp*)	This study
SHQ68	NA1000 Δ*CCNA_03356* (*zapA*)	This study
SHQ69	NA1000 Δ*CCNA_03357* (*zauP*)	This study
SHQ75	SHQ48 *zapA*::*mCherry xylX*::*pHPV472 podJ*::*pHPV489*	This study
SHQ136	SHQ48 *vanA*::*pMT383* (*ftsZyfp*)	This study
SHQ143	NA1000 *zapT*::*mNeonGreen*	This study
SHQ173	SHQ143 *zapA*::*mCherry*	This study
SHQ177	SHQ143 *lacA*::*Pcumate-lacImCherry*	This study
SHQ178	SHQ177 *podJ*::*pHPV489*	This study
UJ9492	NA1000 *3xFLAG-zauP*	This study
UJ9812	NA1000 *zapA*-*3xFLAG*	This study
Escherichia coli		
DH5a	A general cloning strain	Invitrogen
Top10	A general cloning strain	Invitrogen
UJ5191	S17-1 pMT151	42
UJ5195	S17-1 pMT383	42
UJ9399	DH10B pNPTS-ZapA-mCh	This study
UJ9486	DH10B pNPTS-3FzapB	This study
UJ9524	Top10 pQF	65
UJ9727	DH10B pNPTS138-01434_3F	This study
UJ9811	Top10 pNPTSzapA3F	This study
UJ10015	Top10 pNPTSzapA-CKO	This study
UJ10016	Top10 pNPTSzapB-CKO	This study
UJ10032	Top10 pQF01434-3F	This study

**TABLE 2:** Plasmids used in this study^*a*^

Plasmid	Description	Reference or source
mNG-sfTq2	A plasmid with the *mNeonGreen* gene	Addgene
pET21a01434_3F6H	A pET21a (Novagen) derivative to purify ZapT with C-terminal 3xFLAG and 6×His	This study
pLacQF	A pNPTS138 derivative carrying the cumate-dependent promoter between the upstream and downstream regions of the *lacA* gene	This study
pLacQFlacImCherry	A pLacQF derivative with the *lacImCherry* gene	This study
pMT151	A kanamycin resistance-integrating plasmid with the MipZ-YFP ORF under the control of the xylose-dependent promoter	42
pMT383	A kanamycin resistance-integrating plasmid with the FtsZ-YFP ORF under the control of the vanillate-dependent promoter	42
pNPTS-3FzapB	A pNPTS138 derivative with 3F-*zapP*	This study
pNPTS-ZapA-mCh	A pNPTS138 derivative with *zapA*-*mCherry*	This study
pNPTS01434-CKO	A pNPTS138 derivative to introduce an in-frame deletion of *zapT*	This study
pNPTS01434-mNG	A pNPTS138 derivative with *zapT*-*mNeonGreen*	This study
pNPTS138	A kanamycin-resistant suicide vector	66
pNPTS138-01434_3F	A pNPTS138 derivative with *ZapT-*3F	This study
pNPTSzapA-CKO	A pNPTS138 derivative to introduce an in-frame deletion of *zapA*	This study
pNPTSzapA3F	A pNPTS138 derivative with *ZapA-*3F	This study
pNPTSzapB-CKO	A pNPTS138 derivative to introduce an in-frame deletion of *zauP*	This study
pQF	A low-copy-number tetracycline-resistance-conferring vector with the cumate-dependent promoter	65
pQF01434-3F	A pQF derivative with *zapT-3F*	This study
pQFzapTmNG	A pQF derivative with *zapT-mNeonGreen*	This study
pRVCHYC-2	A kanamycin-resistance-conferring plasmid with the mCherry ORF	47

a ORF, open reading frame.

**TABLE 3:** Oligonucleotides used in this study

Oligonucleotide	Sequence (5′–3′)
1	TGTGGTCACCTCGATGTCGG
2	TGACGAACTTCTGCAGGAACA
3	CGAGGTGATCATCGTGTCGAA
4	CAACTATGTCAGCCCGCTCT
5	TATGTAGAGGCGACCCCCAA
6	GGTCTGACGATCCATCACGG
58	TTCCATATGGCGAAGGGGCCAAAC
59	AAAGAATTCTTGTCATCGTCATCCTTGTAATCG
74	CATGCGGAAGCTTCCTCTACTAGTTACAAAC
75	AGTGAGGATCCGGTGAAGTGACCCG
76	GTAACTAGTAGAGGAAGCTTCCGCATGAAACCAGTAACGTTATACGATG
77	CACAGCAGCGGAGCCAGCCGAGCTCGAACCCAGCTGCATTAATGAATCGGCCAAC
78	TTCGAGCTCGGCTGGCTCCGCTGCTGTGAGCAAGGGCGAGGAGGATAACA
79	GGGTCACTTCACCGGATCCTCACTTCTACTTGTACAGCTCGTCCATGCCG
337	GGCGCATGCGTCGTCCGTCTCTAGTTTCAGG
338	ACTCATGTGTCGCTGGGAGAGAGGCGGTACCGCCTCCACCGCGCGCCAAAAGTCCGTCGAGG
339	CCTCGACGGACTTTTGGCGCGCGGTGGAGGCGGTACCGCCTCTCTCCCAGCGACACATGAGT
340	GCAACACCGATTTGGACAGAAATCACTTGTACAGCTCGTCCATGCCCATC
341	GATGGGCATGGACGAGCTGTACAAGTGATTTCTGTCCAAATCGGTGTTGC
342	TCGACTAGTTGATCGTCTCGACCGTCGGCGC
459	AGGAAGCTTCCATATGGCGAAGGGGCCAAACGCCTTCCG
460	GCCGAATTCCGACGCGGAAGGAGCGCCCTTAT
8702	ATAGCATGCGTCGCATGATGGATTTCC
8707	TGCACTAGTCATCATCTTCATGTCACTGCC
8900	AGCGGATCCTTAGTCATCAAGAATAAAAGCAAC
8907	GGCGAATTCCTTGCTGGTGAAGATGCCGGTG
9052	GACGAATTCGATCTATATGTTGCGTCGCATGATG
9053	CCGACCGGTGACGCGTAACGTTCGACTCAGTCGCGAGCTTCTCGATCCGC
9054	GCGGATCGAGAAGCTCGCGACTGAGTCGAACGTTACGCGTCACCGGTCGG
9055	CTTAGTCATCAAGAATAAAAGCAACTACTTGTACAGCTCGTCCATGCCGC
9056	GCGGCATGGACGAGCTGTACAAGTAGTTGCTTTTATTCTTGATGACTAAG
9057	CGCGGATCCACCGATTATTACCTGCCTCGGTCATCAT
9413	ACGGTGATTATAAAGATCATGATATCGATTACAAGGATGACGATGACAAGGGCGGCGGCATCCCGGCCGACAGTACGGCC
9414	TCGTCATCCTTGTAATCGATATCATGATCTTTATAATCACCGTCATGGTCTTTGTAGTCCATCGGCGGAAATCCATCATG
9471	ATTTTCTAGAACGAGCACCCCTACACCG
9472	ATTTGTCGACATCCCGTCGCCGCGGCGC
9950	CTTACTAGTCCGCGCCAAGCGCGCGCGCATG
9951	TGTAATCGATATCATGATCTTTATAATCACCGTCATGGTCTTTGTAGTCGCCGCCGCCACCGCGCGCCAAAAGTCCGTCGAGG
9952	AGACCATGACGGTGATTATAAAGATCATGATATCGATTACAAGGATGACGATGACAAGTGATTTCTGTCCAAATCGGTGTTGC
9953	ACCGCATGCGCGATTCTGTTCCTGGCCGTCC
9954	ATGATTACGCCAAGCTACGTAATACGACTCACTAGTTTCAGGGCACTGGCGCTGCGCGC
9957	ATCCGGAGACGCGTCACGGCCGAAGCTAGCGAATTCCAGCCCGCCCCGCCTGATCCCCGC
10054	TGTAATCGATATCATGATCTTTATAATCACCGTCATGGTCTTTGTAGTCGCCGCCGCCCTCAGTCGCGAGCTTCTCGATCCGC
10055	AGACCATGACGGTGATTATAAAGATCATGATATCGATTACAAGGATGACGATGACAAGTAGTTGCTTTTATTCTTGATGACTA
10220	TGCGGTACCAGGCTGCGCCACGCTCCGACGCG
10461	GGGAAGCTTCCATATGGCGAAGGGGCCAAACGCC
10462	CATCAAGAATAAAAGCAACTACTCAGTCACCTGAGCCATGGATCAGGCCT
10463	AGGCCTGATCCATGGCTCAGGTGACTGAGTAGTTGCTTTTATTCTTGATG
10464	GCTGCGCATCGCCCAATTATCGCCC
10465	TCGAACGGGTCTAAGCCAGACGCGG
10466	GATTTGGACAGAAATCAACCGCGCGCCACCGCAATCGATCCTTAGCCGC
10467	GCGGCTAAGGATCGATTGCGGTGGCGCGCGGTTGATTTCTGTCCAAATC

10.1128/mBio.00487-20.1 TEXT S1 Strains and plasmids used in this study were constructed as described. Download Text S1, DOCX file, 0.02 MB. Copyright © 2020 Ozaki et al. 2020 Ozaki et al. This content is distributed under the terms of the Creative Commons Attribution 4.0 International license.

### Microscopy.

Differential interference contrast, phase-contrast, and fluorescence microscopy analyses were performed using a Nikon Eclipse 80i microscope and an Andor Zyla 4.2 scientific complementary metal oxide semiconductor (sCMOS) camera. Quantitative image analyses of cells and fluorescent signals were performed using the Oufti and MicrobeJ software packages (58, 59).

For visualization of the nucleoid, cells were fixed in 70% ethanol, harvested by centrifugation, and resuspended in phosphate-buffered saline containing 2 μg/ml DAPI (4′,6-diamidino-2-phenylindole).

### FACS.

The fluorescence-activated cell sorter (FACS) assay was performed essentially as described previously (60–62). In brief, exponentially growing cells (100 μl) were fixed in ice-cold 70% ethanol and then harvested by centrifugation and washed in 1 ml of FACS buffer (10 mM Tris-HCl [pH 7.5] and 1 mM magnesium chloride). The DNA was stained in 1 ml of FACS buffer supplemented with 2 μM Sytox Green (Thermo Fisher Scientific) at room temperature for 0.5 h. The fluorescent intensity and light scattering were analyzed using a FACSCalibur system (BD Biosciences).

### CoIP-MS.

To perform immunoprecipitation, an overnight culture (100 ml) was diluted in fresh PYE medium (1 liter) and cultivated at 30°C for 3 h. The cells were harvested by centrifugation (5,000 rpm, 20 min, 4°C) and resuspended in IP wash buffer (10 mM HEPES KOH [pH 7.4] and 75 mM sodium chloride) to adjust the optical density at 600 nm (OD_600_) to 100 (9 ml cell suspension). After the addition of cOmplete protease inhibitor (Roche) and 25 μg/ml DNase I, the cells were lysed through two passages in a French press. The sample was then incubated at 4°C for a further 15 min in the presence of 0.1% IGEPAL, followed by ultracentrifugation (30,000 rpm, 1 h, 4°C). The supernatant was mixed with a 30-μl slurry of anti-FLAG M2 magnetic beads (Sigma-Aldrich) equilibrated with buffer (10 mM HEPES KOH [pH 7.5], 75 mM NaCl, and 0.1% IGEPAL) and was incubated for 1 h at 4°C with gentle agitation. The beads were harvested using a magnetic stand and washed three times in 700 μl of 0.1 M ammonium bicarbonate. Bead-bound materials were eluted in two steps using (i) 100 μl of elution buffer 1 (0.1 M ammonium bicarbonate, 1.6 M urea, 0.5 μg trypsin) and (ii) 80 μl of elution buffer 2 [0.1 M ammonium bicarbonate, 1.6 M urea, 0.2 M Tris(2-carboxyethyl)phosphine]. Liquid chromatography-tandem mass spectrometry was performed as described previously (61).

### ChIP-seq.

ChIP was performed essentially as described previously (61). Briefly, exponentially growing cells were fixed for 10 min using a 1% formaldehyde solution, washed thoroughly, resuspended in buffer, and lysed through two passages in a French press. After DNA shearing with sonication was performed, the cell debris was removed by ultracentrifugation and the cleared cell lysate was incubated with anti-FLAG M2 magnetic beads (Sigma-Aldrich). After washing of the beads was performed, the bound materials were incubated at 65°C overnight to reverse cross-linking. The resulting DNA samples were purified using a DNA cleanup kit (Macherey-Nagel) and then analyzed by deep sequencing (single-end NextSeq).

The sequence data were analyzed using Galaxy (https://usegalaxy.eu). Briefly, Bowtie 2 was used to map the reads onto the NA1000 reference genome. After read extension to 300 nucleotides (nt), the coverage of every 50-bp window was deduced. The values normalized to the total number of reads were used to identify the ChIP peaks. Two independent ChIP-seq experiments were performed to confirm the reproducibility of the results.

### Purification of ZapT-His.

Rosetta 2(DE3) cells (Novagen) harboring pET21a01434_3F6H were grown exponentially in LB medium (1 liter) supplemented with ampicillin and chloramphenicol, and ZapT-His was induced for 3 h by addition of 1 mM isopropyl-β-d-1-thiogalactoside. The resulting cells were harvested by centrifugation and resuspended in buffer A (25 mM Tris HCl [pH 7.5], 300 mM sodium chloride, 5% glycerol) supplemented with 5 mM imidazole and 0.2 mg/ml lysozyme. The cell suspension was incubated on ice for 30 min, frozen in liquid nitrogen, and thawed to lyse cells. After ultracentrifugation (48,000 rpm, 20 min), the supernatant was loaded onto a nickel-Sepharose 6 Fast Flow column (0.5 ml) equilibrated with buffer A containing 5 mM imidazole. After washing was performed with buffer A containing 20 mM imidazole, the ZapT-His proteins retained on the column were eluted in buffer A containing 250 mM imidazole. ZapT-His was further purified using HiTrap heparin (1 ml): ZapT-His was loaded in buffer B (25 mM Tris HCl [pH 7.5], 5% glycerol) containing 100 mM NaCl, washed in buffer B (plus 200 mM sodium chloride) and in buffer B (plus 500 mM sodium chloride), and eluted in buffer B (plus 2,000 mM sodium chloride). After dialysis against buffer B (plus 100 mM sodium chloride), 0.22 mg/ml of ZapT-His (0.8 ml) was obtained (see Fig. S3 in the supplemental material).

### Electrophoretic mobility shift assay (EMSA).

This assay was performed essentially as described previously (63, 64). Ligand DNA (10 nM) and ZapT-His were incubated for 10 min on ice in 10 μl of buffer (20 mM HEPES KOH [pH 7.6], 100 mM sodium chloride, 10% glycerol, 0.01% Triton X-100, 0.1 mg/ml bovine serum albumin, 5 mM magnesium acetate). The samples were analyzed using 6% polyacrylamide gel electrophoresis, and DNA was visualized by GelStar (Lonza) staining. Ligands E1, E2, and E3 were generated by PCR using NA1000 and primers 1 and 2 for E1, primers 3 and 4 for E2, and primers 5 and 6 for E3.
