# Supplementary figures and images for: Novel Divisome-Associated Protein Spatially Coupling the Z-Ring with the Chromosomal Replication Terminus in Caulobacter crescentus
Source: mBio. 2020 Apr 28;11(2):e00487-20. doi: 10.1128/mBio.00487-20 (PMC7188993; doi:10.1128/mBio.00487-20)

Supplemental Figure S1

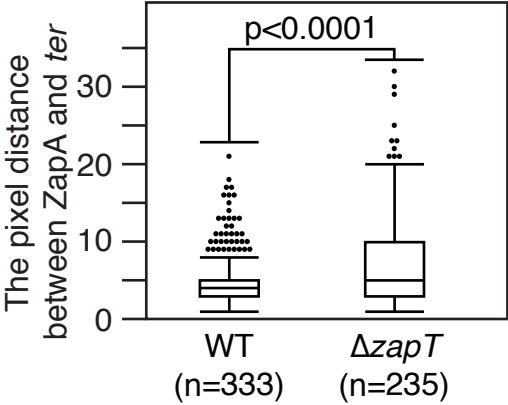

Supplement: FIG S1 [file mBio.00487-20-sf001.pdf]

# Supplemental Figure S2

A

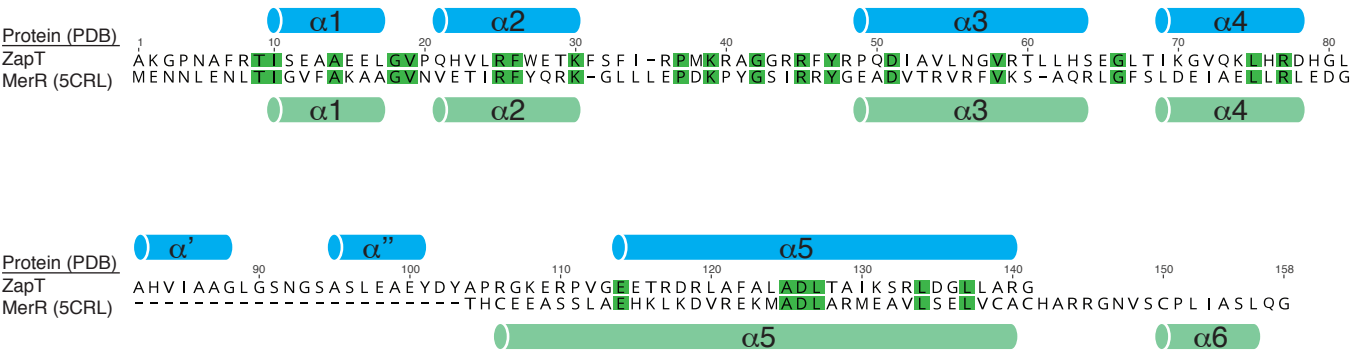

B

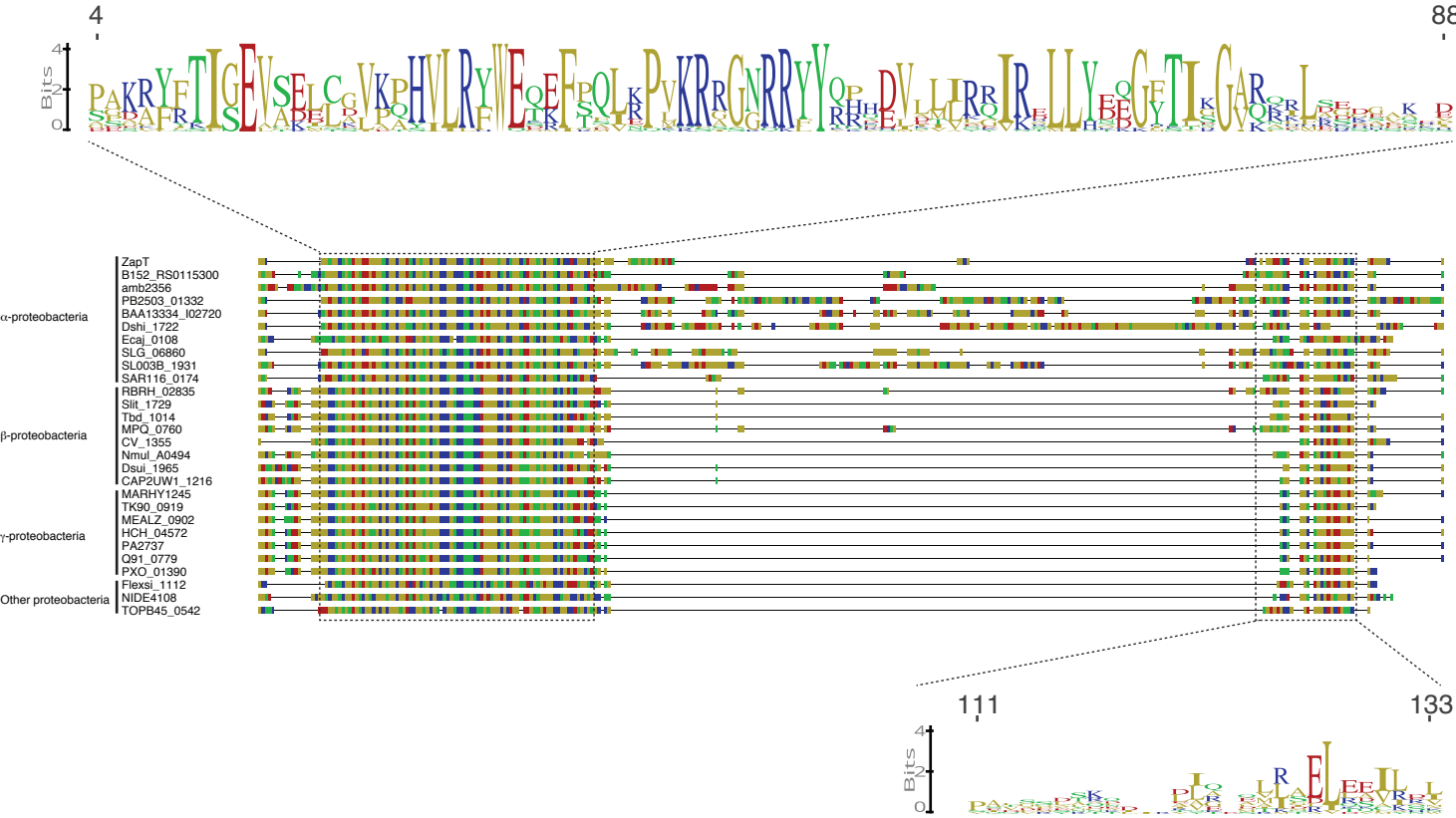

Supplement: FIG S2 [file mBio.00487-20-sf002.pdf]

Supplemental Figure S3

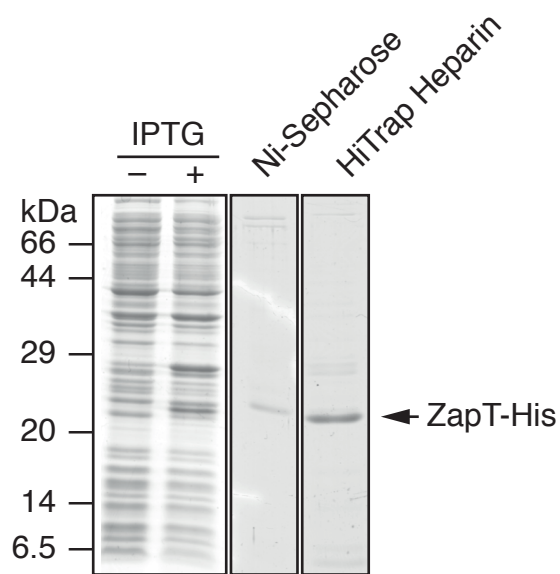

Supplement: FIG S3 [file mBio.00487-20-sf003.pdf]

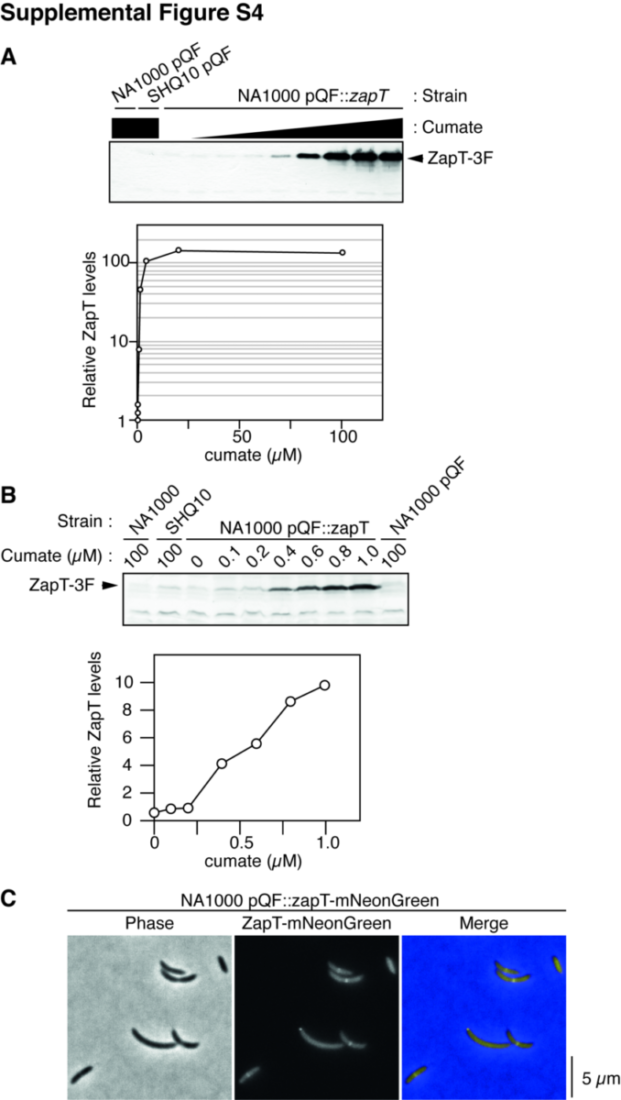

Supplement: FIG S4 [file mBio.00487-20-sf004.tif]

Supplemental Figure S5

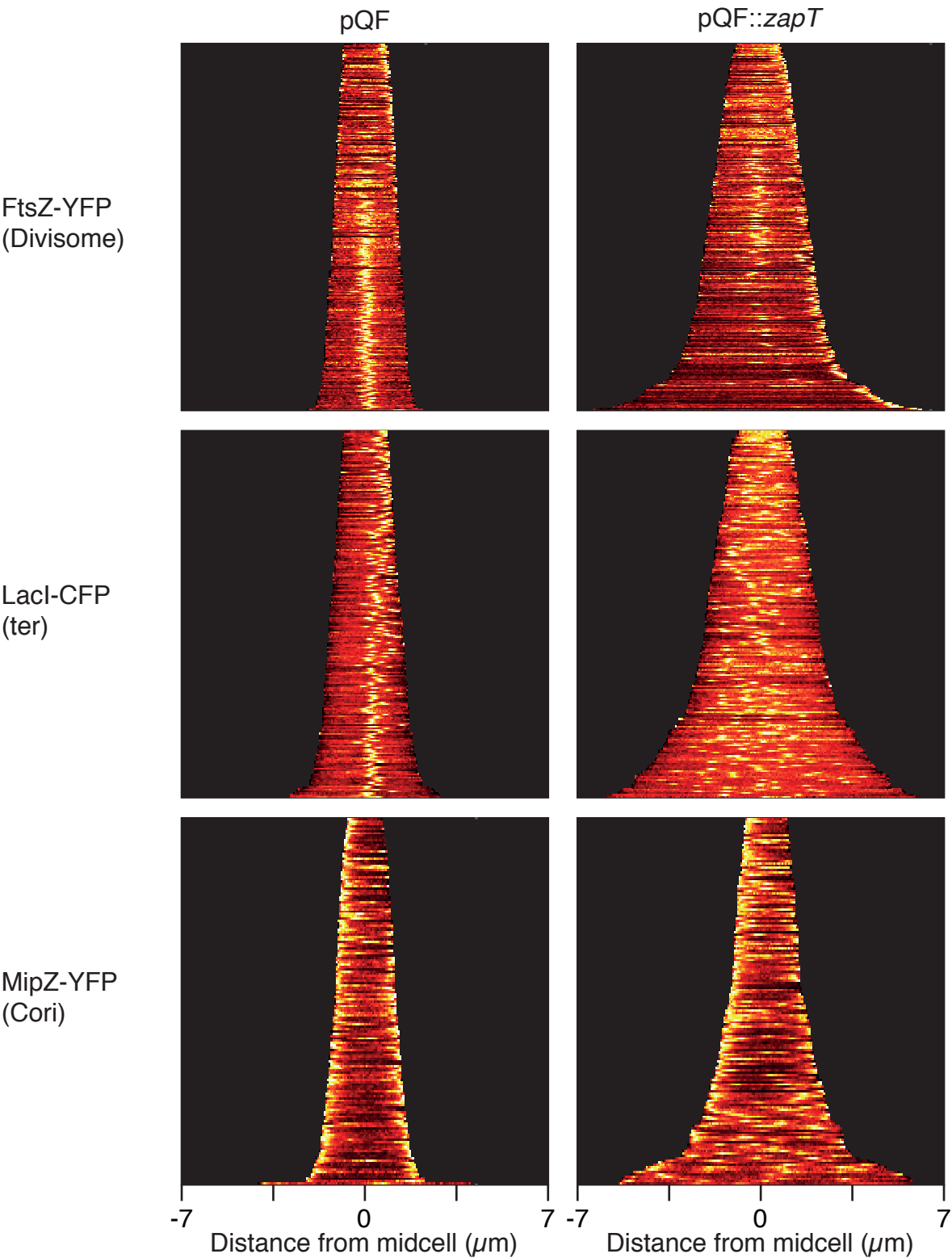

Supplement: FIG S5 [file mBio.00487-20-sf005.pdf]

Supplemental Figure S6

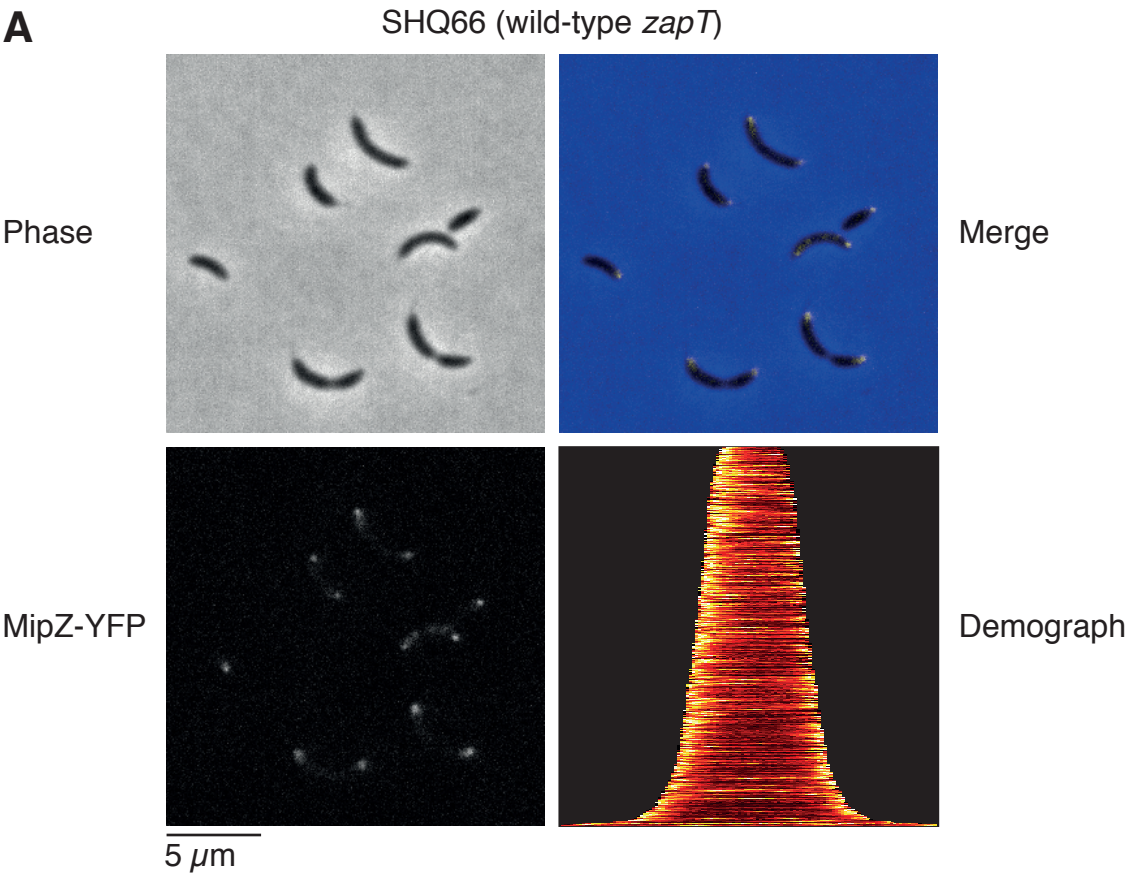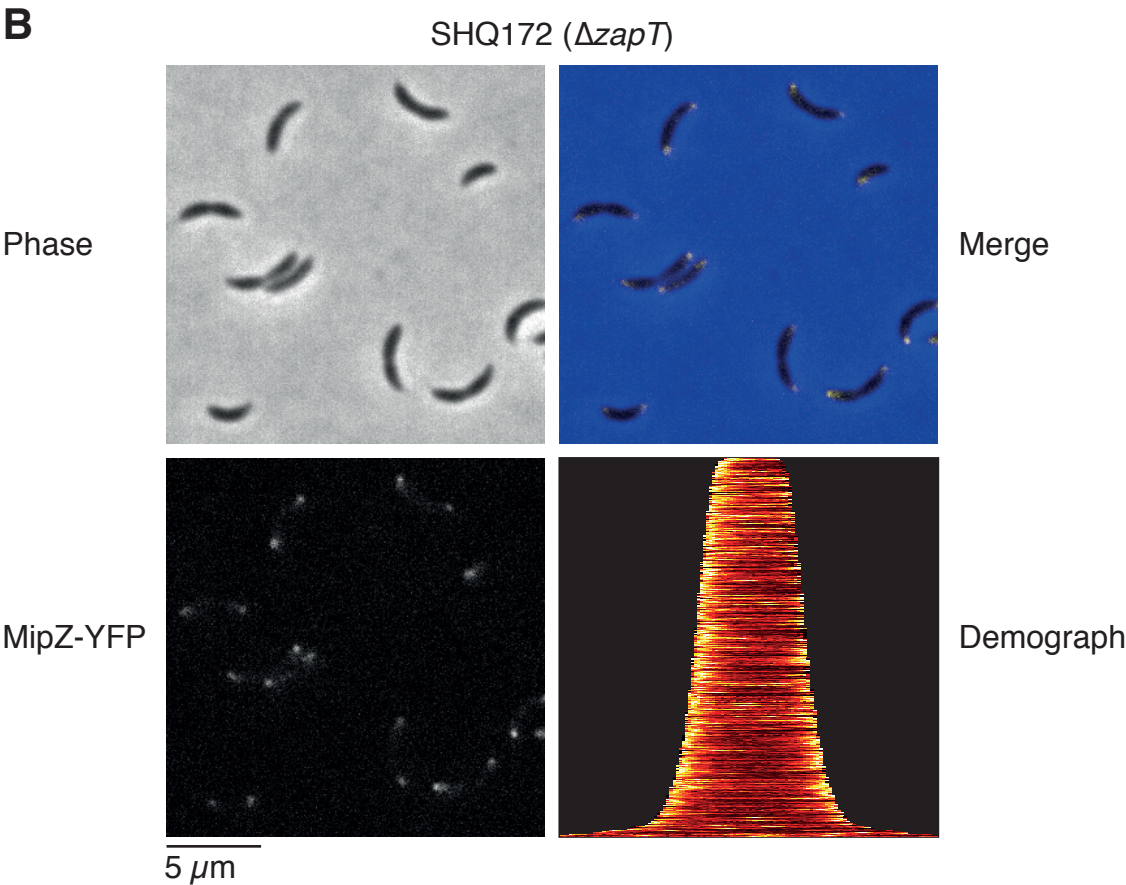

Supplement: FIG S6 [file mBio.00487-20-sf006.pdf]
