# Supplementary material for: Novel Divisome-Associated Protein Spatially Coupling the Z-Ring with the Chromosomal Replication Terminus in Caulobacter crescentus
Source: mBio. 2020 Apr 28;11(2):e00487-20. doi: 10.1128/mBio.00487-20 (PMC7188993; doi:10.1128/mBio.00487-20)
Supplement: TEXT S1 [file mBio.00487-20-s0001.docx]

**Supplemental Material**

**Bacterial strains**

The SHQ10, SHQ48, SHQ56, SHQ68, SHQ69, SHQ143, SHQ173, SHQ177, and UJ9492, and UJ9812 strains were generated by double homologous recombination using pNPTS138-01434_3F, pNPTS01434-CKO, pNPTS-zapAmCh, pNPTSzapA-CKO, pNPTSzapB-CKO, pNPTS01434-mNG, pNPTS-zapAmCh, pLacQFlacImCherry, pNPTS-3FzapB, and pNPTSzapA3F, respectively. The SHQ63, SHQ75, and SHQ178 strains were constructed by sequential two-step transduction using a Cr30 phage lysate of PV2865.

To construct the SHQ66 strain, pMT151 was introduced into the NA1000 strain via electroporation. Integration of the plasmid was verified by PCR. The SHQ67 and SHQ136 strains were constructed similarly by transformation with pMT383.

**DNAs**

To construct pNPTS138-01434_3F, a 635 bp DNA was amplified by PCR using NA1000 genomic DNA and primers 9950/9951. In addition, a 571 bp product was amplified by PCR using NA1000 genomic DNA and primers 9952/9953. The two fragments were combined by overlap extension PCR with primers 9950/9953, yielding a 1165 bp insert DNA. After digestion with SpeI and SphI, the insert was ligated to the SpeI-SphI fragment of pNPTS138.

To construct pNPTS01434-CKO, a 593 bp DNA was amplified by PCR using NA1000 genomic DNA and primers 9954/10466. In addition, a 514 bp DNA was amplified by PCR using NA1000 genomic DNA and primers 10467/9957. The two fragments were combined by overlap extension PCR with primers 9954/9957, yielding a 1058 bp insert DNA. After digestion with SpeI and EcoRI, the inset was ligated to the SpeI-EcoRI fragment of pNPTS138.

To construct pNPTS-zapAmCh, a 2 kb insert DNA was generated by overlap extension PCR. First, a 0.78 kb DNA was amplified by PCR using NA1000 genomic DNA and primers 9052/9053. Second, 0.79 kb DNA was amplified from pRVCHYC-2 template using primers 9054/9055. Third, a 0.52 kb DNA was amplified using NA1000 genomic DNA and primers 9056/9057. The three fragments were combined by overlap extension PCR with primers 9052/9057. After digestion with EcoRI and BamHI, the products were ligated to the EcoRI-BamHI fragment of pNPTS138.

To construct pNPTS-3FzapB, an 802 bp DNA was amplified by PCR using NA1000 genomic DNA and primers 8900/9413. In addition, a 601 bp DNA was amplified by PCR using NA1000 genomic DNA and primers 8907/9414. The two fragments were combined by overlap extension PCR with primers 8900/8907, yielding a 1360 bp insert. After digestion with EcoRI and BamHI, the inset was ligated to the EcoRI-BamHI fragment of pNPTS138.

To construct pNPTSzapA3F, an 814 bp DNA was amplified by PCR using NA1000 genomic DNA and primers 9052/10054. In addition, a 554 bp DNA was amplified by PCR using NA1000 genomic DNA and primers 10055/9057. The two fragments were combined by overlap extension PCR with primers 9052/9057, yielding a 1327 bp insert. After digestion with EcoRI and BamHI, the inset was ligated to the EcoRI-BamHI fragment of pNPTS138.

To construct pNPTSzapA-CKO, a derivative of pNPTS138 containing the ∆*zapA*(∆6-107 aa) allele and the flanking region, a 457 bp DNA was amplified by PCR using NA1000 genomic DNA and primers 8702/10462. In addition, a 503 bp DNA was amplified by PCR using NA1000 genomic DNA and primers 10463/8707. The two fragments were combined by overlap extension PCR with primers 8702/8707, yielding a 910 bp insert. After digestion with SpeI and SphI, the inset was ligated to the SpeI-SphI fragment of pNPTS138.

To construct pNPTSzapB-CKO, a derivative of pNPTS138 containing the ∆*zauP* (∆21-87 aa) allele and the flanking region, a 629 bp DNA was amplified by PCR using NA1000 genomic DNA and primers 8900/10464. In addition, a 505 bp DNA was amplified by PCR using NA1000 genomic DNA and primers 10465/8907. The two fragments were combined by overlap extension PCR with primers 8900/8907, yielding a 1084 bp insert. After digestion with BamHI and EcoRI, the inset was ligated to the BamHI-EcoRI fragment of pNPTS138.

To construct pQF01434-3F, a 604 bp insert DNA was amplified by PCR using pNPTS138-01434_3F and primers 10461/10220. After digestion with HindIII and KpnI, the inset was ligated to the HindIII-KpnI fragment of pQF.

To construct pET21a01434_3F6H, a 512 bp insert DNA was amplified by PCR using pNPTS01434_3F and primers 58/59. After digestion with NdeI and EcoRI, he inset was ligated to the NdeI-EcoRI fragment of pET21a (Novagen).

To construct pLacQF, a DNA fragment containing a part of the *C. crescentus* *lacA* gene was amplified by PCR using NA1000 genome and primers 9471/9472. The product was digested with XbaI and SalI and ligated to the SpeI-SalI fragment of pNPTS138, yielding a vector DNA. Then, pQF was digested with PciI, blunted using Klenow frgagment, followed by EcoRI digestion to release an insert DNA containing *cymR** and P_Q5_. The insert was ligated to the SmaI-EcoRI fragment of the vector DNA.

To construct pLacQFlacImCherry, a 1.1 kb DNA was amplified by PCR using *E. coli* MG1655 genome and primers 76/77. In addition, a 0.76 kb DNA was amplified by PCR using pNPTS-zapAmCh and primers 78/79. The two fragments were combined by overlap extension PCR with 76/79, yielding a 1.8 kb insert. The vector pLacQF was linearized by PCR using primers 74/75. The insert and the vector were digested with SpeI and EcoRI, and then ligated to each other.

To construct pNPTS01434-mNG, a 2.2 kb insert DNA was generated by overlap extension PCR. First, a 1013 bp DNA was amplified by PCR using NA1000 and primers 337/338. Second, a 740 bp DNA was amplified by PCR using sfTq2-mNG (addgene) and primers 339/340. Third, a 558 bp DNA was amplified by PCR using NA1000 and primers 341/342. The three fragments were combined by overlap extension PCR with primers 337/342. After digestion with SpeI and SphI, the products were ligated to the SpeI-SphI fragment of pNPTS138.

To construct pQFzapTmNG, a 1203 bp insert DNA was amplified by PCR using pNPTS01434-mNG and primers 459/460. After digestion with HindIII and EcoRI, the inset was ligated to the HindIII-EcoRI fragment of pQF.
